# Supplementary material for: Sick without signs. Subclinical infections reduce local movements, alter habitat selection, and cause demographic shifts
Source: Commun Biol. 2024 Nov 1;7:1426. doi: 10.1038/s42003-024-07114-4 (PMC11530534; doi:10.1038/s42003-024-07114-4)
Supplement: Supplementary file 2 — Supplementary Information [file 42003_2024_7114_MOESM2_ESM.pdf]

Supplementary Materials for 'Sick without signs. Subclinical infections reduce local movements, alter habitat selection, and cause demographic shifts'

Marius Grabow*, Wiebke Ullmann, Conny Landgraf, Rahel Sollmann, Carolin Scholz, Ran Nathan, Sivan Toledo, Renke Luehken, Joerns Fickel, Florian Jeltsch, Niels Blaum, Viktoriia Radchuk, Ralph Tiedemann, Stephanie Kramer-Schadt

*Corresponding author: Marius Grabow; email: grabow@izw-berlin.de

Content:

S1 (movie) Exemplary movement tracks

S2 Environmental variables

S3 Animal capturing

S4 Laboratory protocols

S5 Pre-processing of animal tracking data

S6 autocorrelated Kernel Density Estimate (aKDE)

S7 Behavioural classification

S8 Subsampling to 30-minute resolution

S9 Habitat selection

S10 Morphological responses

S11 Candidate Multievent models

S12 Estimates of selected Multievent models

S13 Evidence in favour of lower survival in infected individuals

S14 Multievent capture-mark-recapture (MECMR) simulations

S1 (movie): Exemplary movement tracks

Animation to show movement data of swallows during foraging events in the study area. Each dot represents an individual swallow on a foraging trip, and each line indicates the suggested path between two recorded points. Due to computational constraints, the original tracking data were resampled from 8-second recordings (0.125Hz) to 40-second intervals. Note: Resampling increases the temporal resolution by a factor of five, displayed here solely for illustrative purposes.

Data collection

S2 Environmental variables

All environmental variables are based on colour-infrared biotope types [1], which we subsequently processed for further analyses. Based on the biotope types, we first calculated Euclidean distance raster layers of a) distance to waterbodies, b) distance to kettle holes, c) distance to human settlements, d) distance to streets, e) distance to rivers, and f) distance to the capture location utilizing *terra* [2]. At each animal's location, we extracted these covariates to assess the correlation among environmental factors. To avoid multicollinearity, we excluded any covariates from the same model where the absolute of Pearson's moment correlation coefficient ($|r|$) was greater than 0.6. We used *corrplot* [3] to visualize results (Figure F1).

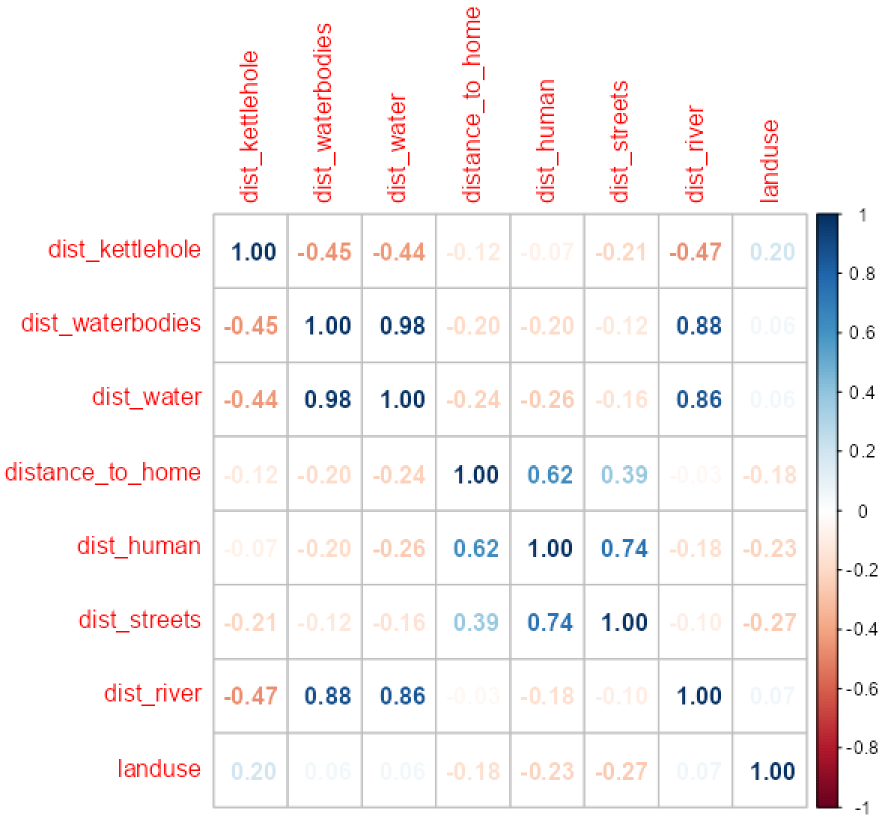

F1: Correlation matrix of all environmental covariates extracted at each animal localisation

Secondly, to streamline our analyses, we consolidated the detailed habitat types to only four habitat classes, capturing the essential characteristics of each habitat types while avoiding excessive detail (Table T1).

Table T1: Consolidated habitat types used in analysis based on detailed habitat mapping from aerial scans

Consolidated habitat type	Detailed habitat types based on the classification by LfU Brandenburg [1]
Human	Gardens & graveyards (10XXXXXX)<br>Special biotope types (11XXXXXX)<br>Built up areas (12XXXXXX)
Agricultural	Agricultural (9XXXXXX)
Semi-natural	Bushland (7XXXXXX)<br>Forests (8XXXXXX)
Water-related	Flowing water (1XXXXXX)<br>Standing water (2XXXXXX)

	Ruderal areas (3XXXXXX)<br>Swamps (4XXXXXX)<br>Grassland (5XXXXXX)

S3 Animal capturing

In 2019, we performed a pilot study that was not restrictive on capturing effort and sampling schemes, violating assumptions of capture mark-recapture (cmr) models and thus excluded from demography models and the tracking study. However, blood sampling few individuals (Barn swallows: n=20; House martins: n=3) revealed a blood parasite prevalence of 33% in house martins, and 5% in barn swallows. During capture, we equipped swallows with ATLAS tags <1 g (Figure F2) and observed flight behaviour after releasing the animals and controlled if they return to their colony.

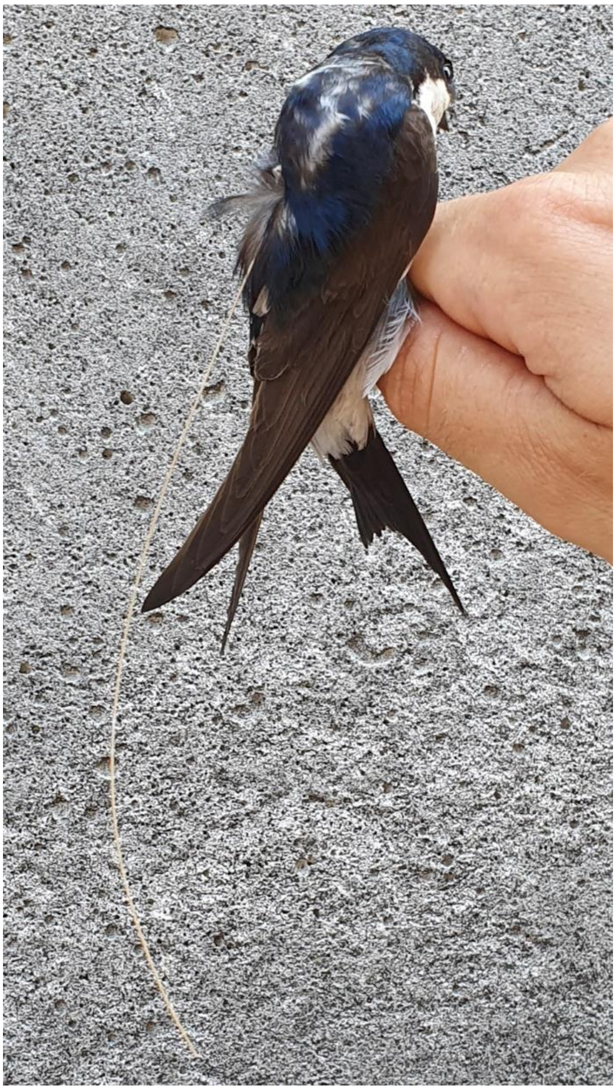

Figure F2: House martin (HM) with ATLAS tag

S4 Laboratory protocols

To evaluate the presence of infections, DNA was isolated and purified from whole blood samples using spin-column kits, followed by a nested PCR process employing primer pairs as specified by Hellgren et al. [4], with the first nested PCR using HaemNF1/HaemNR3 and the second nested PCR using HaemF/HaemR2. The resulting PCR fragments were examined on a 2% agarose gel stained to detect the presence of DNA, where positive reactions were indicated by a distinct band around 500 base pairs. In 2023 we changed from non-heparinized to sodium-heparinized capillaries for the blood collection. To assess if PCR protocols could be inhibited by heparin, we performed additional sex-PCR by using the CHD1F/ CHD1R primer to amplify fragments of the CHD gene [5,6], and found no differences between both blood collection methods ($\chi^2(1, N = 230) = 1.21$, $p = 0.271$).

Animal tracking (ATLAS)

S5 Pre-processing of animal tracking data

We pre-processed all raw ATLAS tracking data before utilizing it for movement models, behavioural classification and microhabitat selection analyses. First, we performed basic spatial data analyses, such as projecting coordinates and obtaining geographic coordinates (latitude, longitude) using the *sf* package [7,8] and the *dplyr* package [9]. We used a covariance filter (COVXY < $2500m^2$) and speed filters (speed < $30ms^{-1}$) to remove localisations with high spatial uncertainty or highly unrealistic movement speeds. Subsequently, we removed all observations between dawn and nautical dusk (i.e. sun position = 12°) using the *suncalc* package and the exact spatial position of the individual to include all flights shortly before sunset or after sunrise [10]. We realigned timestamps of individual observations, ensuring a consistent sampling rate of 8 seconds (0.125Hz) and removed duplicate observations. To guarantee reliable continuous time movement models (ctmm), we removed individuals with fewer than 500 observations, which typically indicated tag losses shortly after the tagging events. Similarly, we removed the first six hours after the tagging to reduce biases regarding the handling of the animals and the cmr study (i.e., opened mist-nets). For handling missing data, we used a dual-strategy approach depending on the length of the data gap: For short gaps, defined as less than 25 consecutive missing observations (equivalent to 3.73 minutes ≈ 25 × 8 seconds), we utilized the *ctmm* package [11] to predict and reconstruct the path based on actual locations and the estimated movement model. Typically, this missing data occurs because single observations during movement were missing, for example when swallows forage very close to the ground, making it challenging to detect the signal send by the tags. For larger gaps, usually occurring during 'resting' behaviour when animals are stationary (as observed in our swallow colonies in the dairy farm, where the ATLAS signal could not be retrieved), we manually adjusted the data. We did this by drawing

random points within 10 × 10 m around the last observed location, ensuring minimal movements aligning with the typical ATLAS error of few meters [12].

Individual responses to parasites

S6 autocorrelated Kernel Density Estimate (aKDE)

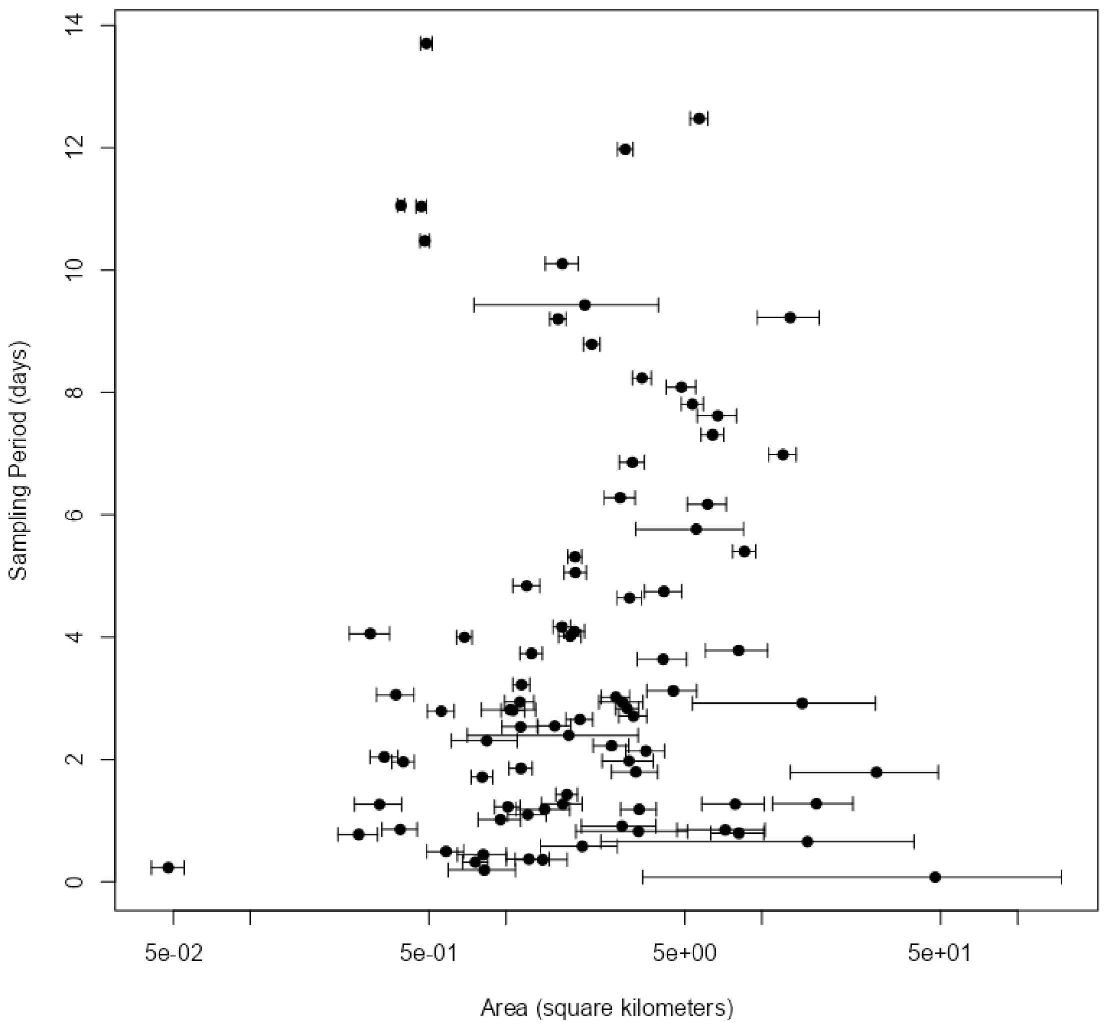

Figure F3: Funnel plot of 95% AKDE size in relation to sampling period (days)

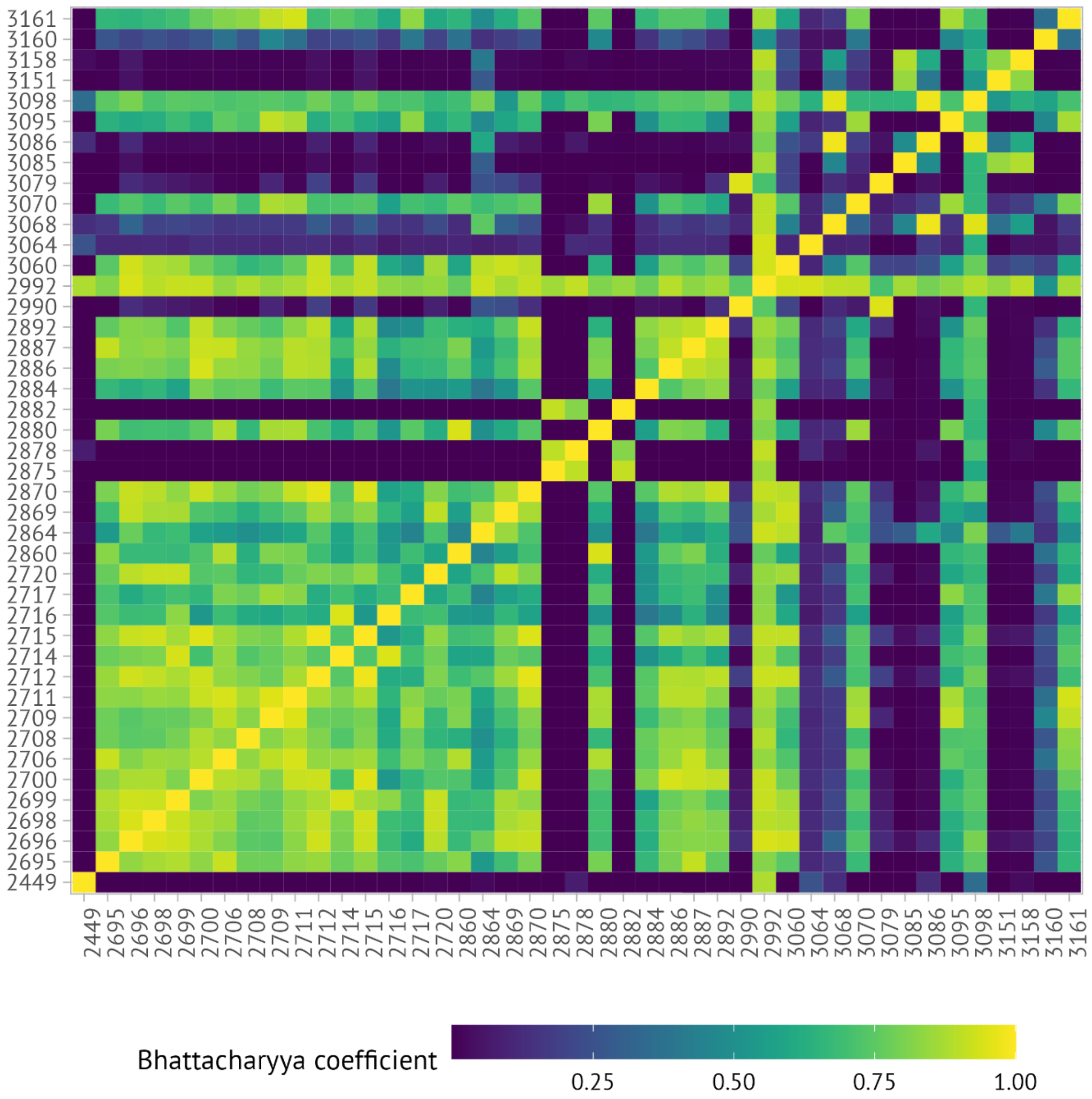

Figure F4: Visualisation of spatial overlap in tagged individuals using the Bthattacharyya coefficient [13]

S7 Behavioural classification

Barn swallow:

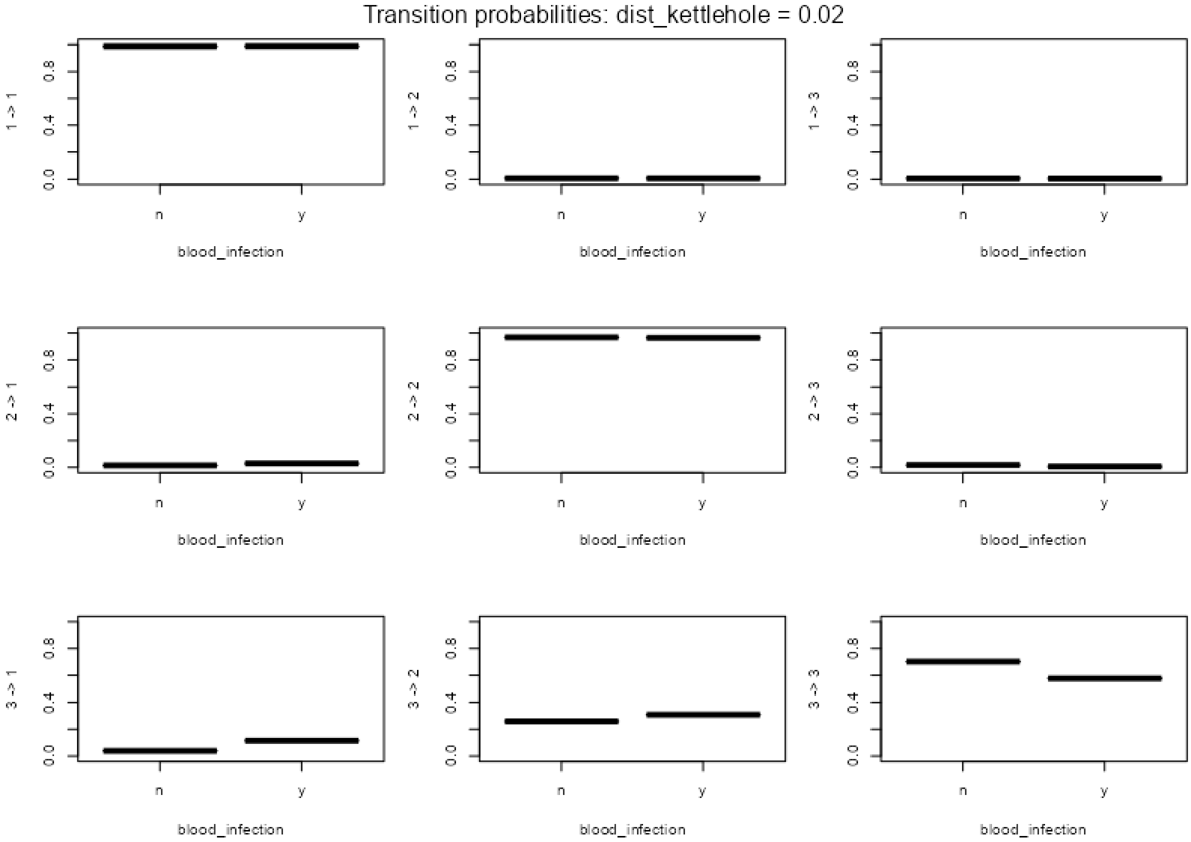

Figure F5: State transition probabilities (barn swallows); states: 1= resting, 2=foraging, 3= commuting

House martin:

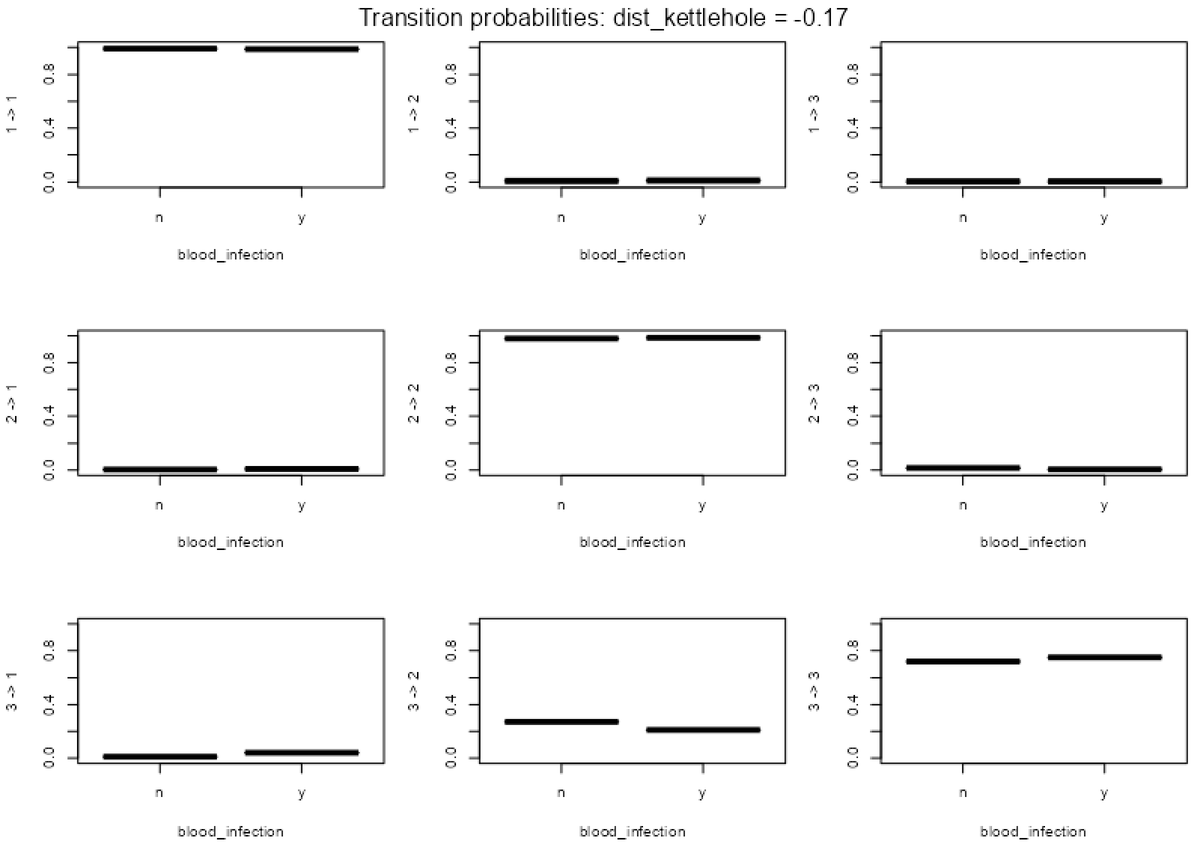

Figure F6: State transition probabilities (house martins); states: 1= resting, 2=foraging, 3= commuting

S8 Subsampling to 30 minute resolution

We tested if we could reveal the same behavioural differences between infected and non-infected individuals if we did not use high-resolution movement data. Therefore, we resampled our data to 30 min resolution (Figure F7) and choose initial parameters (step length and turning angle) for the HMMs aligning with the spatiotemporal scale. We were unable to reliably detect behavioural states (Figure F8-F10), hence could not identify the foraging locations, which would be required to study habitat selection during foraging behaviour.

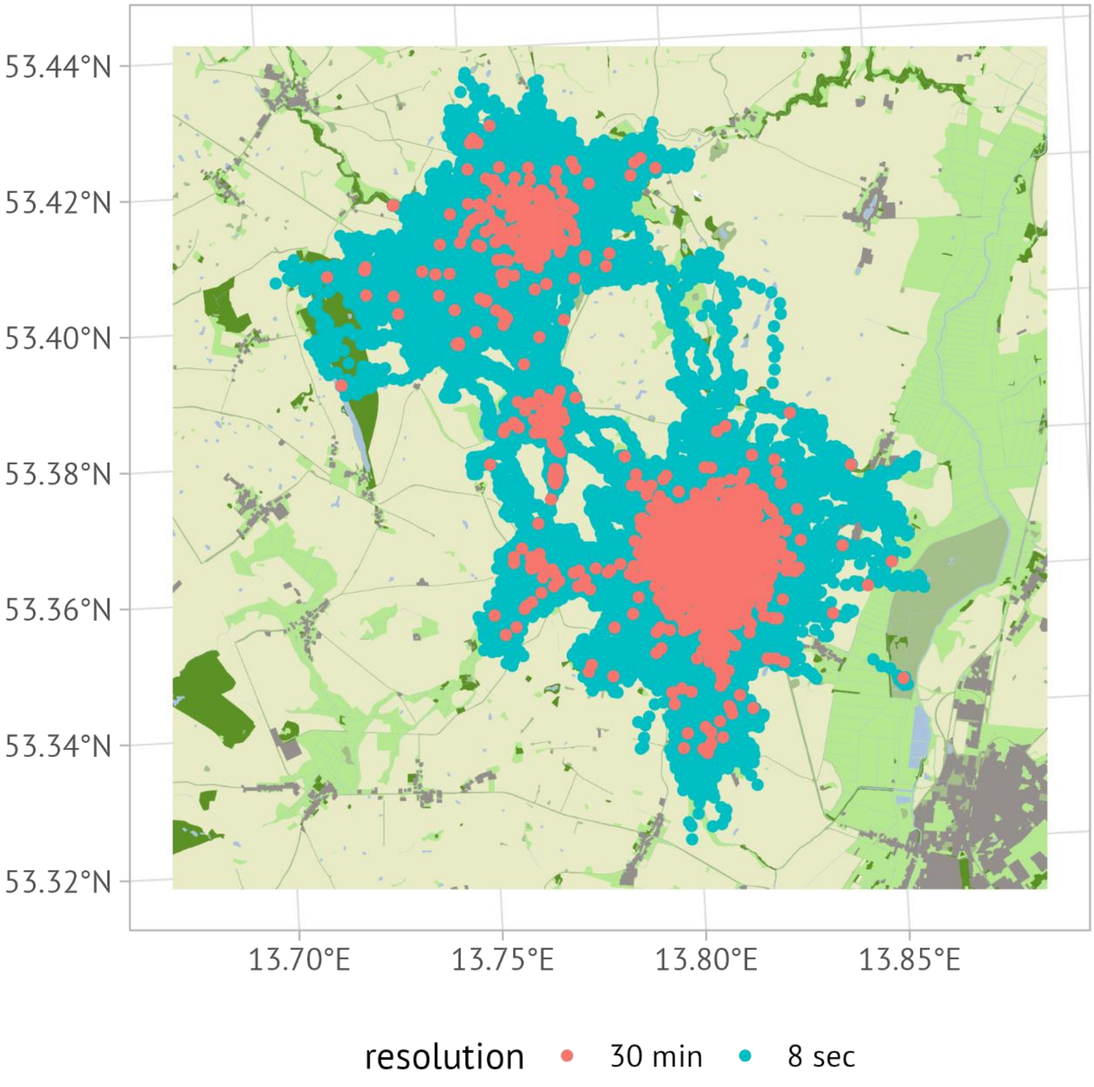

Figure F7: Visual comparison of subsampled data to a resolution of 30 minutes to the high-resolution movement data of 8 seconds

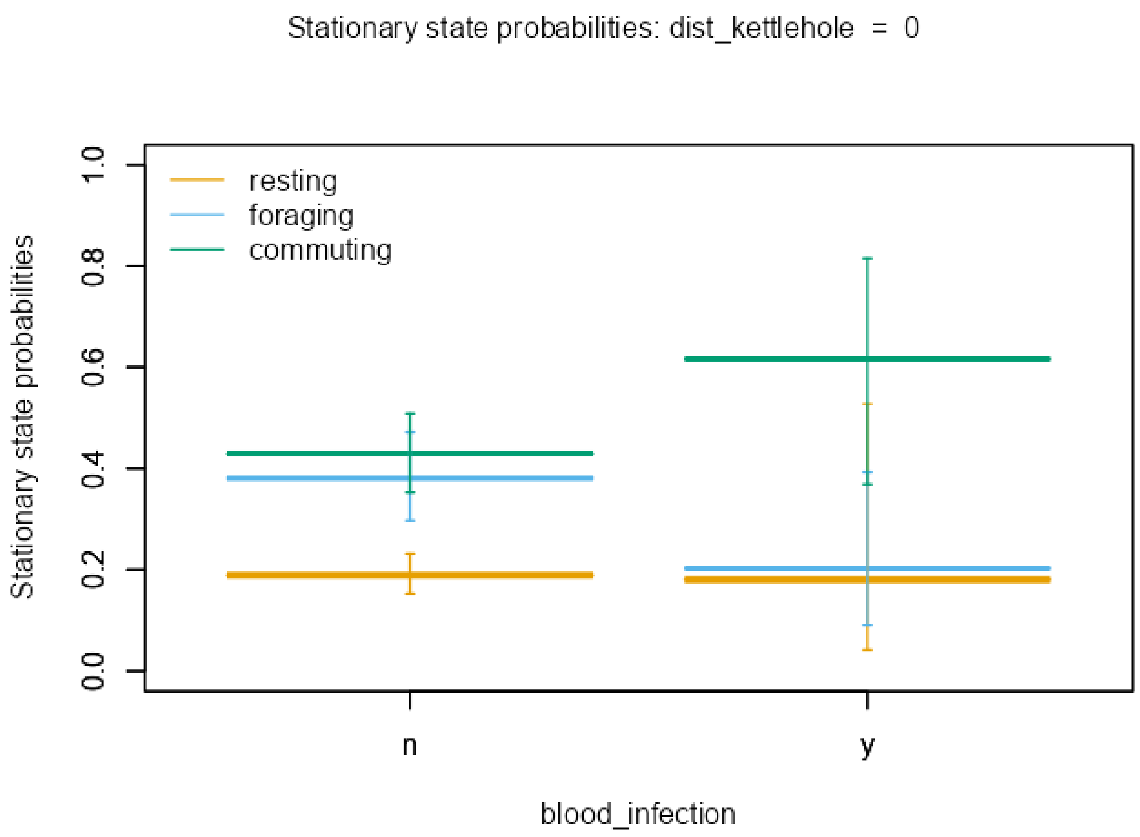

Figure F8: Stationary states probabilities of non-infected and infected barn swallows (BS). Please note that this is resampled data (30 minutes) that fails to detect behavioural states.

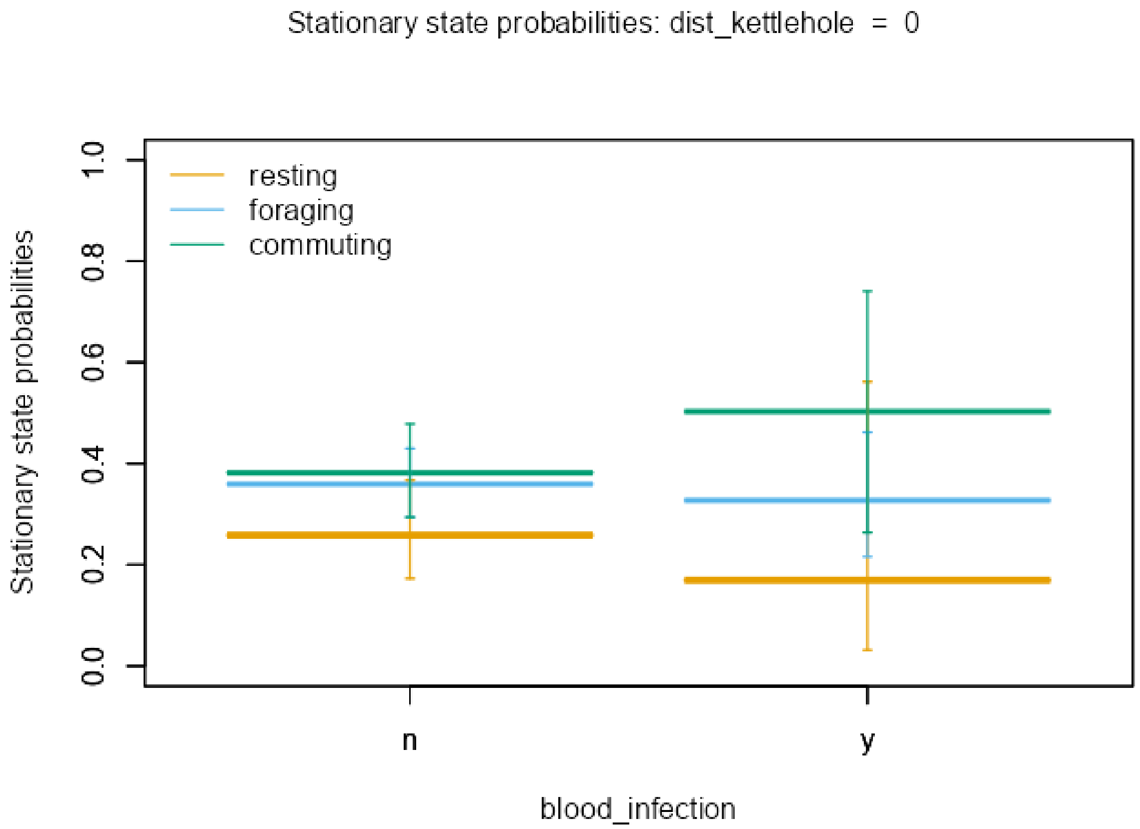

Figure F9: Stationary states probabilities of non-infected and infected house martin (HM). Please note that this is resampled data (30 minutes) that fails to detect behavioural states.

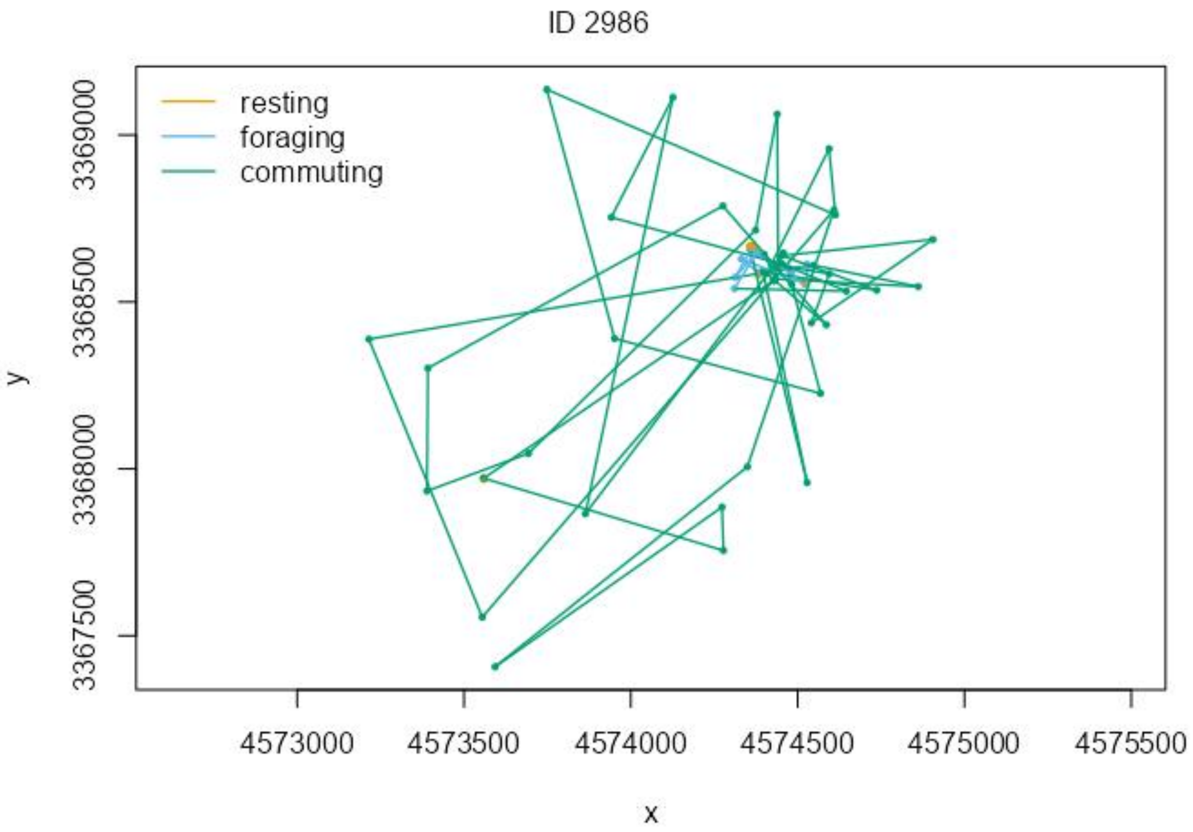

Figure F10: Exemplary classification of one movement track. Please note that this is resampled data (30 minutes) that fails to detect behavioural states. While longer resting periods around the colony are still detected (see resting), all movement states are diluted and especially foraging states are not recognized as such.

S9 Habitat selection

After fitting integrated step selection functions [14] on the behavioural state of 'foraging' that was previously identified by the Hidden-Markov Model (HMM), we fitted the same step selection function to all movement data, i.e. ignoring the underlying state. We did this to circumvent potential misclassifications by the HMM and tested if the same effects would occur if we would ignore the behavioural states. We fitted a second integrated step selection function using the same model formula on the full data set. Here, we could verify that infected individuals indeed used agricultural landscapes more frequently compared to non-infected individuals (Figure F11).

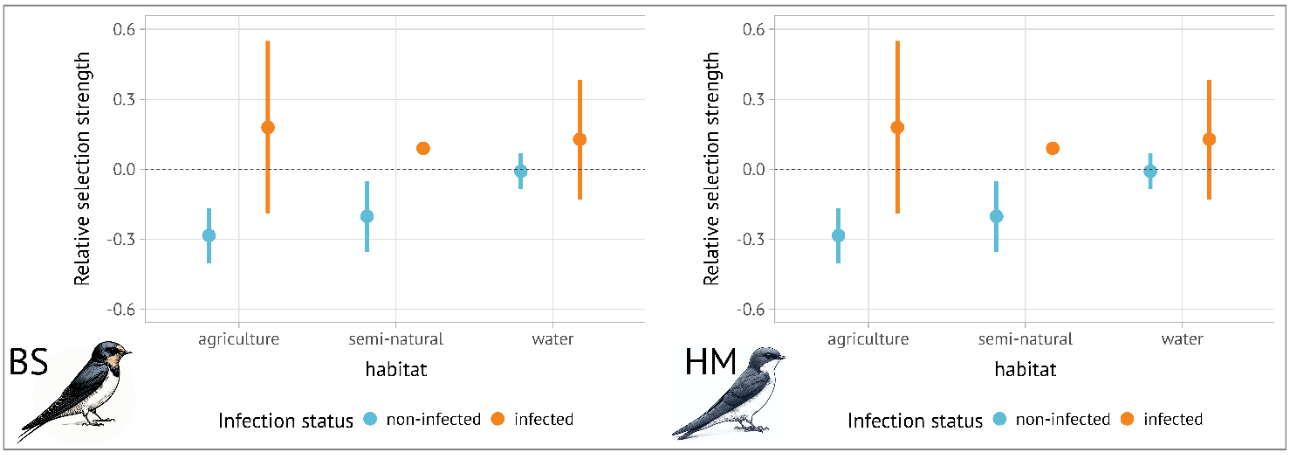

Figure F11: Repeated Integrated Step selection function analysis fitted on all movement data (including resting and commuting)

S10 Morphological responses

We performed Principal component analyses (PCA) on all body traits that we collected during the capture-mark-recapture study, omitting all body traits that we only collected for less than 60% of the population. We utilized PCA via the R-package *stats* and visualised results using the *factoextra* package [15]. We used bi-plots and overlaid the results of a PCA with the categorical variable reflecting the infection status to control if body traits were unrelated to infection status.

S10.1 Barn swallow

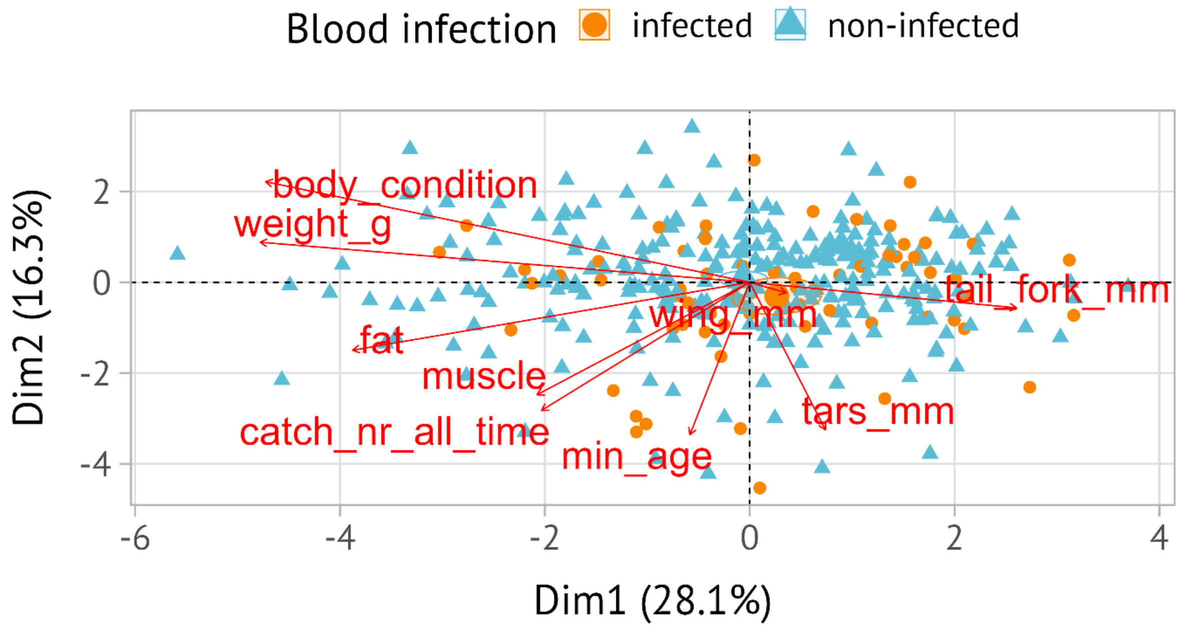

Figure F12: Principal component analysis (PCA) bi-plot on all body traits of barn swallows (BS). Blood infection status was overlaid to visualize results.

S10.2 House martin

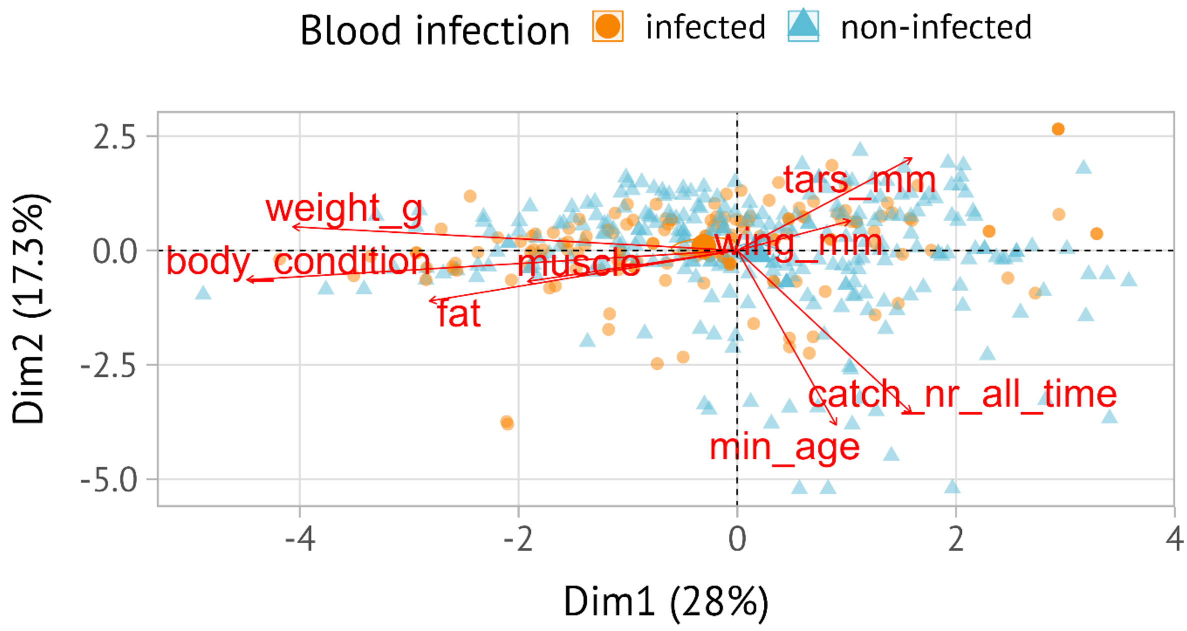

Figure F13: Principal component analysis (PCA) bi-plot on all body traits of house martin (HM). Blood infection status was overlaid to visualize results.

S10.3 Morphological trait changes during active parasitic infection

We calculated the difference in body condition (body weight in g / tarsometatarsus length in mm) for each individual that was recaptured in the recapturing, within 14 days after the initial capturing and excluded all individuals if we lacked parasite infection data We found both increases and decreases in individual body condition of both species and in both infection statuses. (Figure F14). In non-infected barn swallows body conditions decreased over the period of 14 days on average by -0.004g mm^{-1} ± 0.139 (mean ± SD), in infected barn swallows body conditions increased by 0.103 g mm^{-1} ± 0.118 (mean ± SD). In contrast, in non-infected house martins body conditions increased by 0.037 mm^{-1} ± 0.148 (mean ± SD), and decreased in infected house martins by -0.023mm^{-1} ± 0.121 (mean ± SD). Effects were neither statistically significant in barn swallows (Welch t-test: t(7.5946) = -2.777, p =0.081), nor in house martins (Welch t-test: t(60) = 1.776, p =0.081).

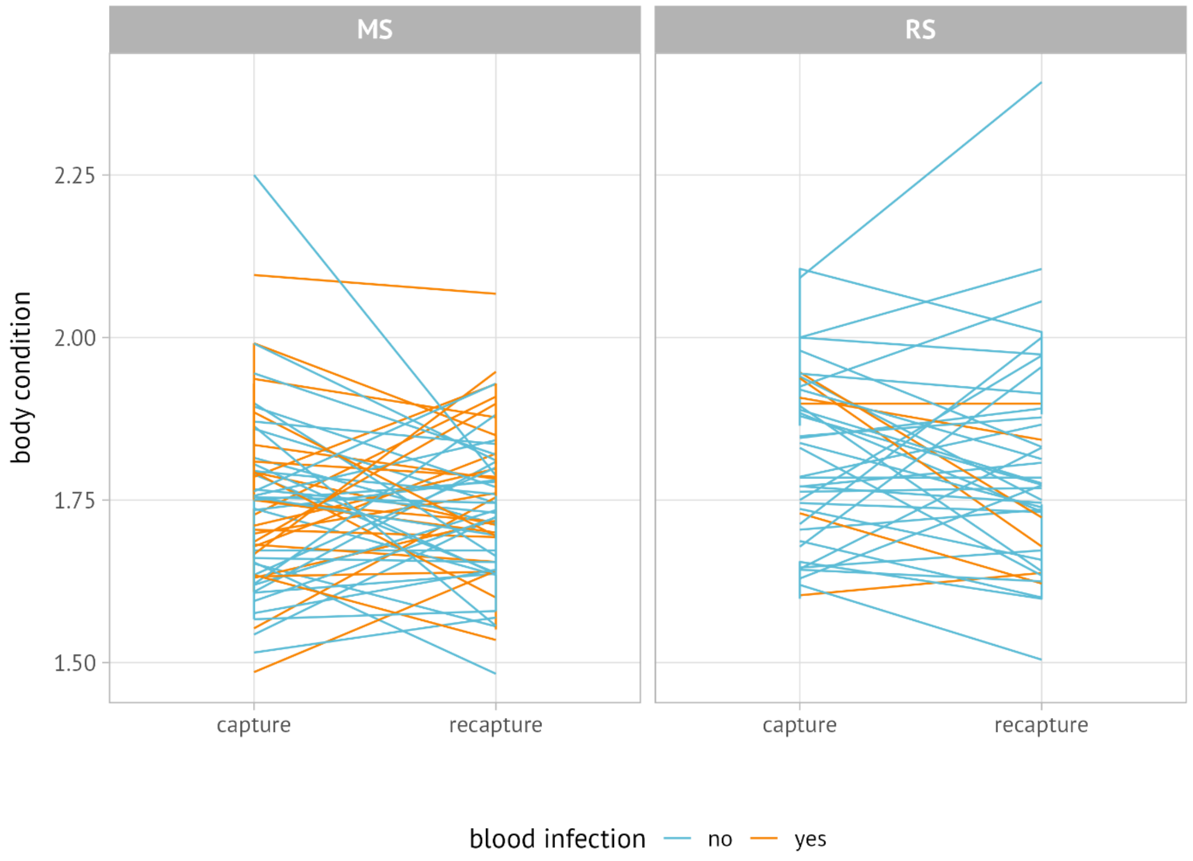

Figure F14: Changes in body condition across one CMR sampling interval. Each line represents an individual with its changes in body condition across one CMR sampling interval, i.e. lines with a negative slope indicate a decrease in body condition, and lines with a positive slope indicate an increase in body condition.

Demographic responses

S11 Candidate Multi-event models

We evaluated various demographic models for barn swallows (BS) and house martins (HM), aiming to primarily detect the effects of parasites on survival probability (ϕ) and additionally testing for other covariates that could influence survival probabilities. All models used fixed detection probabilities for non-infected (p_{neg}) and infected (p_{pos}) individuals. All models include prior infection status of the previous year (infection t-1). Subsequently, we added individual covariates that are not time-dependent, which do not change over time (M2: sex, M3: tarsus). Additionally, we tested time-dependent individual covariates (M4: prior infection, M8: body condition, M9 sex and body condition, M10: body condition in interaction with infection status), and time-dependent population covariates (M5: disease prevalence within the colony). Model performance is assessed through the Weighted Akaike Information Criterion (WAIC) and ΔWAIC indicating the relative performance against the best model (Table T2).

Table T2: Candidate models for demography models for barn swallows (BS) and house martins (HM). Please note that missing models M6 and M7 violated model assumptions and are left out intentionally

Species	Model	Formula ϕ	pWAIC	WAIC	ΔWAIC
BS	**M8**	**ϕ ~ infection $_{t-1}$ + body condition$_{t-1}$**	**38.814**	**1435.837**	-
	M4	ϕ$_t$ ~ infection $_{t-1}$	39.459	1440.426	4.599
	M2	ϕ ~ sex + infection $_{t-1}$	39.874	1508.245	72.416
	M3	ϕ ~ sex + infection $_{t-1}$ * tarsus	38.855	1737.118	301.281
	M9	ϕ ~ infection $_{t-1}$ + sex * body condition $_{t-1}$	39.505	1756.257	321.960
	M1	ϕ ~ infection	39.763	2130.355	696.058
	M10	ϕ ~ infection $_{t-1}$ * body condition $_{t-1}$	35.507	2250.732	814.895
	M5	ϕ ~ infection$_{t-1}$ * prevalence $_{t-1}$	39.992	2836.208	1401.911

HM	**M4**	**ϕ_t ~ infection $_{t-1}$**	**50.332**	**1192.365**	**-**
	M1	ϕ ~ infection	48.953	1201.139	8.773
	M8	ϕ ~ infection $_{t-1}$ + body condition$_{t-1}$	49.339	1227.872	35.507
	M10	ϕ ~ infection $_{t-1}$ * body condition $_{t-1}$	48.668	1426.444	234.078
	M5	ϕ ~ infection$_{t-1}$ * prevalence $_{t-1}$	50.126	1461.212	268.847
	M3	ϕ ~ sex + infection $_{t-1}$ * tarsus	47.991	1587.016	394.651
	M9	ϕ ~ infection $_{t-1}$ + sex * body condition $_{t-1}$	49.263	1610.048	417.683
	M2	ϕ ~ sex + infection $_{t-1}$	49.440	1780.352	587.986

S12 Estimates of selected Multievent models

Model selection yielded different models per species (Supplementary material S9), indicating that models including body conditions describes barn swallow population dynamics best; while models with varying survival probabilities between years described the house martin population dynamics best (Table T3).

Table T3: Parameter estimates after model selection for barn swallow (BS) and house martin (HM).

Parameter	Barn swallow (BS)	House martin (HM)
Survival non-infected<br>($\Phi_{t\ \text{non-infected}}$)	0.481 [89% CI: 0.383-0.585]	2020: 0.557 [89% CI: 0.301-0.811]<br>2021: 0.468 [89% CI: 0.280-0.659]<br>2022: 0.340 [89% CI: 0.204-0.490]
Survival infected<br>($\Phi_{t\ \text{infected}}$)	0.429 [89% CI: 0.270-0.622]	2020: 0.333 [89% CI: 0.092-0.622]<br>2021: 0.359 [89% CI: 0.212-0.515]<br>2022: 0.375 [89% CI: 0.253-0.510]
Detection probability non-infected<br>($p_{non\text{-}infected}$)	0.343 [89% CI: 0.295-0.389]	0.638 [89% CI: 0.577-0.701]
Detection probability infected<br>($p_{\text{-}infected}$)	0.212 [89% CI: 0.140-0.315]	0.537 [89% CI: 0.461-0.613]
Transition probability infection Ψ_{negpos}	0.262 [89% CI: 0.123-0.412]	0.438 [89% CI: 0.262-0.607]
Transition probability clearing infection* Ψ_{posneg}<br>*Detected by PCR, not considering chronic infection	0.579 [89% CI: 0.168-0.855]	0.569 [89% CI: 0.387-0.754]

Probability being infected upon first capture (π_{first})	0.742 [89% CI: 0.685-0.800].	0.551 [89% CI: 0.478-0.628]

S13 Evidence in favour of lower survival in infected individuals

To assess the evidence for lower survival in infected individuals, we calculated the difference between all posterior samples from model posterior distributions. Specifically, we combined posterior samples from multiple chains, then computed the difference between the survival probabilities of infected and non-infected individuals. The resulting distribution of these differences allowed us to estimate the proportion of samples with lower survival in infected individuals. We visualized this distribution using density plots, focusing on the proportion of the distribution that falls below zero, which reflects evidence in favour of reduced survival in infected individuals

S13.1 Barn swallow

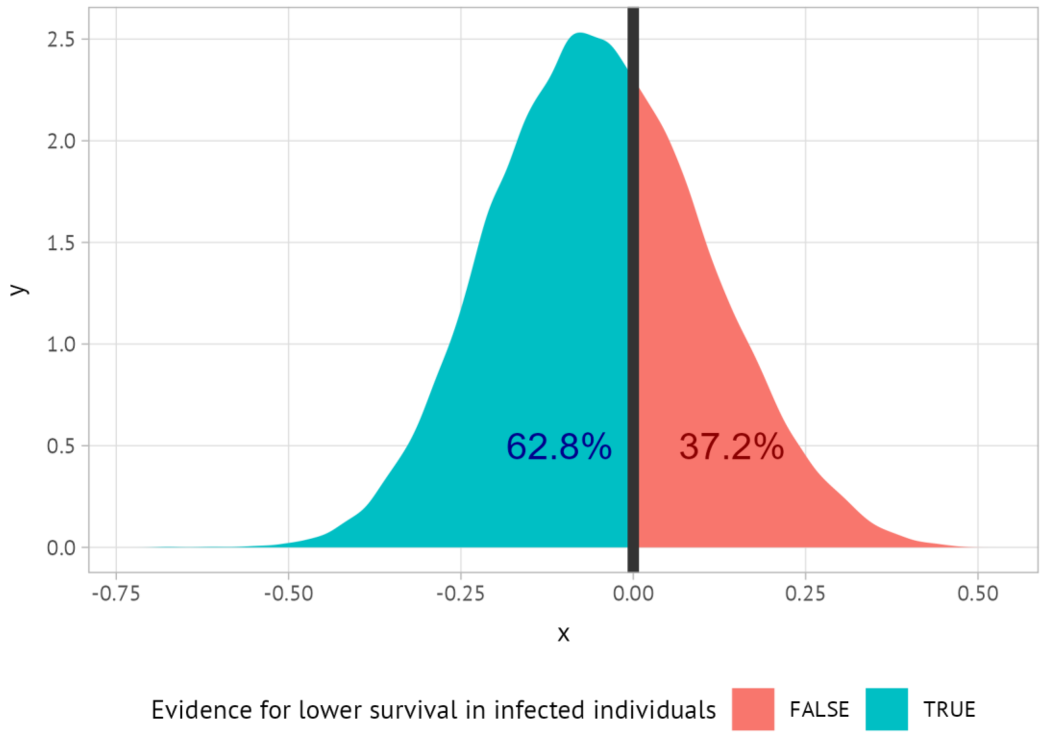

Figure F15: Evidence in favour for lower survival of infected individuals in barn swallows (BS)

S13.2 House martin

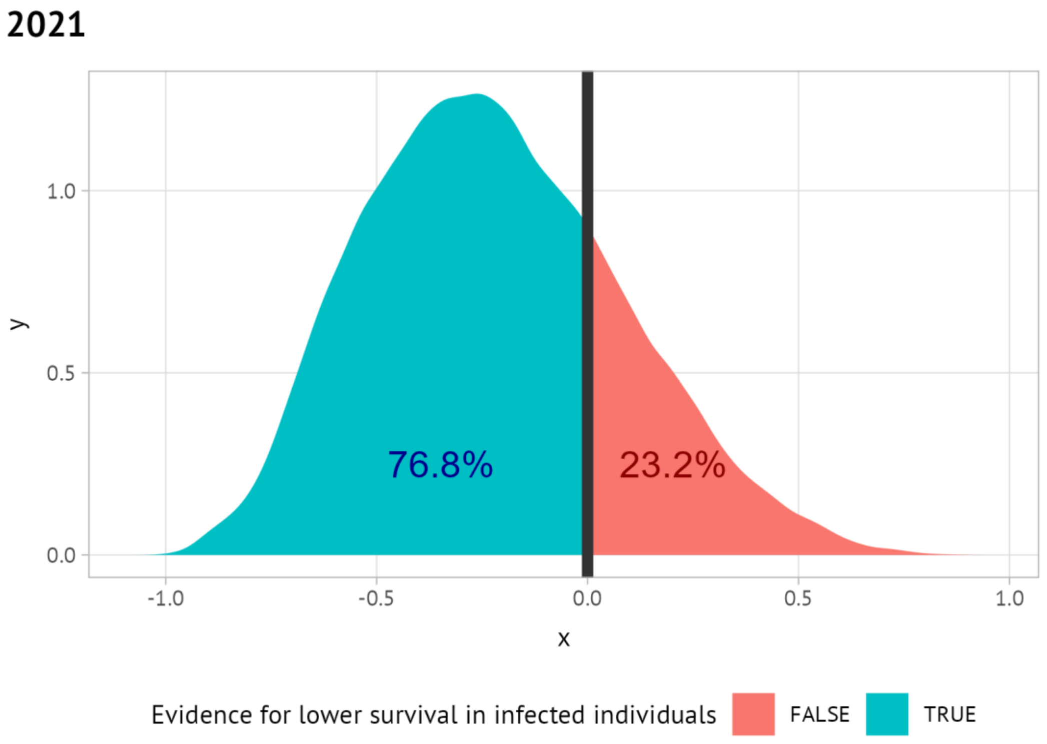

Figure F16: Evidence in favour for lower survival of infected individuals in house martins (HM) in 2021

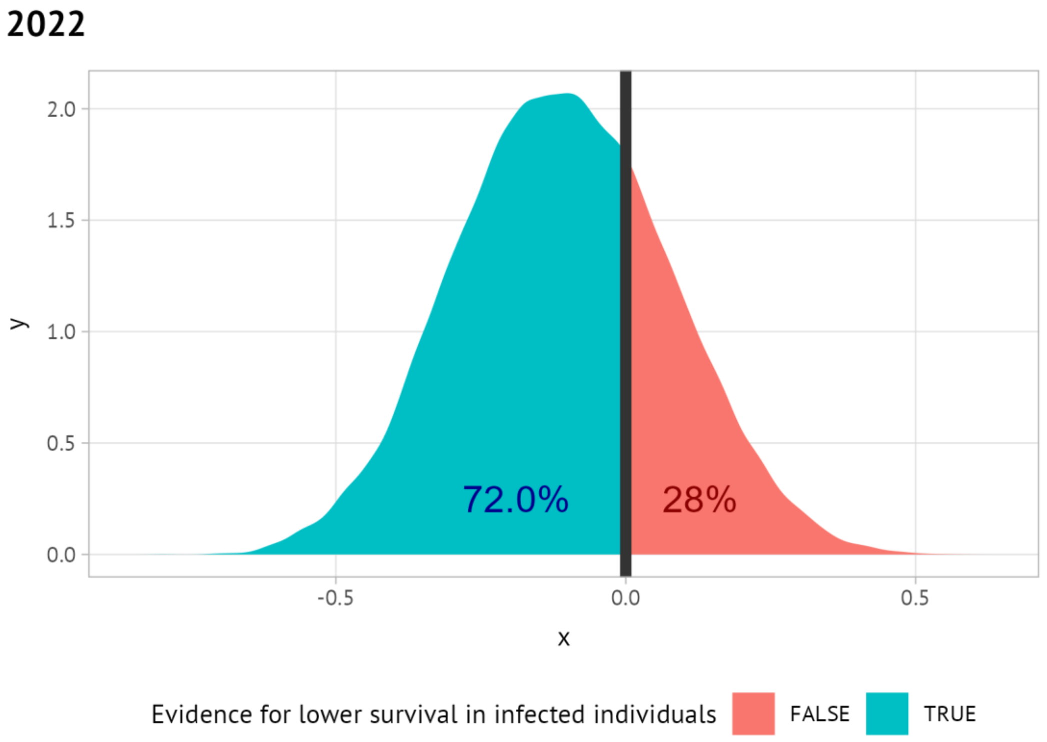

Figure F17: Evidence in favour for lower survival of infected individuals in house martins (HM) in 2022

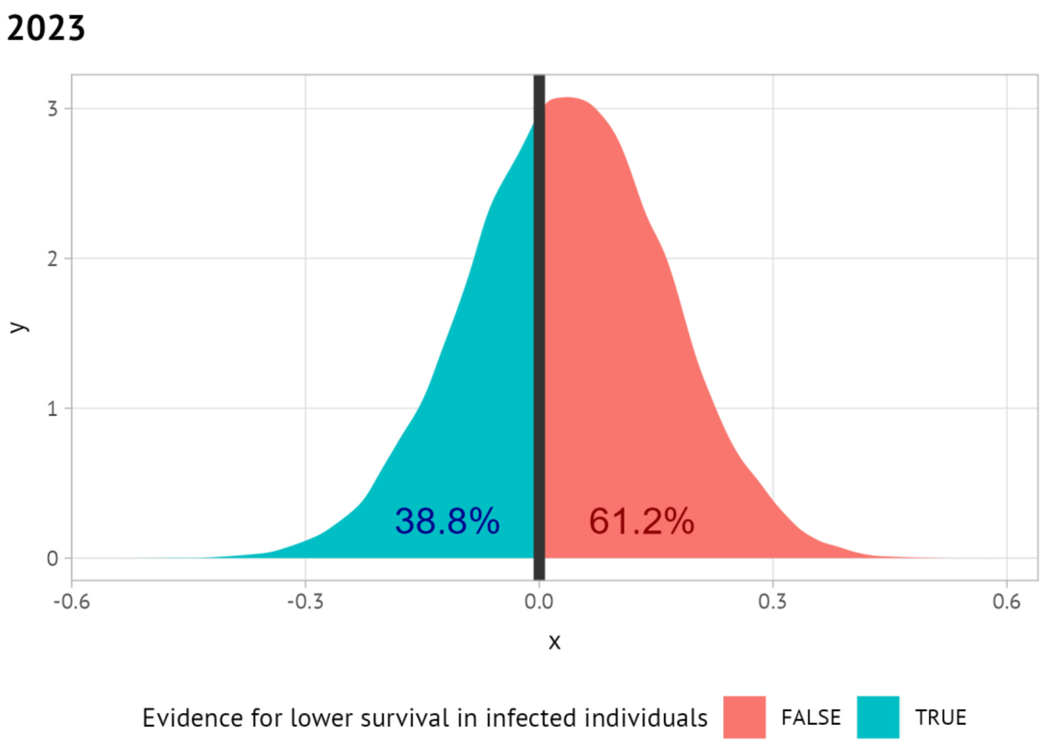

Figure F18: Evidence in favour for lower survival of infected individuals in house martins (HM) in 2023

S14 Multievent capture-mark-recapture (MECMR) simulations

We designed individual capture histories that accurately mirrored the number of sampling occasions and sampling sizes inherent to our study system to assess if our sample sizes are sufficient to receive unbiased estimates. Therefore, we simulated our study system by simulating random 'true' survival values for infected and non-infected individuals. Specifically, we simulated individual capture histories for 385 individuals, over 4 years of capturing, utilizing 2 secondary capture occasions. Next, we configured the model parameters related to probabilities, specifically calibrating survival, detection, state transition, testing, and first-year capture prevalence for both infected and non-infected individuals. We did this by drawing random numbers from uniform distributions, excluding biologically un-meaningful values around 0 and 1 for survival, detection, and testing probabilities (Table T4).

Table T4: Parameter intervals for which we took random numbers from uniform distributions over an interval from the minimum value to the maximum value.

Parameter	min. value	max. value
$\phi_{BP_{neg}}$	0.2	0.8
$\phi_{BP_{pos}}$	0.2	0.8
p_{neg}	0.2	0.8
p_{pos}	0.2	0.8
β_{Test}	0.5	1
$\psi_{BP_{negpos}}$	0	1
$\psi_{BP_{posneg}}$	0	1
π_1	0	1

Using these probabilities, we formulated an individual state probability matrix that accommodates non-detections based on the given capture probabilities and incorporates the modelled testing probability. Similarly, we established a transition probability matrix for the three distinct states: alive and infected, alive and non-infected, and dead. For the initial capture state of a first-year individual, which could only be infected or non-infected, we employed a binomial distribution with our predetermined probabilities. Following this, we generated multinomially distributed random numbers based on our state probability and transition probability matrices. We iterated this process 50 times per model (i.e. 100 survival estimates, 50 survival estimates for infected and 50 survival estimates for non-infected individuals). Subsequently, we assessed the accuracy with which our

model identified the ‘true’ survival probabilities by assessing if the parameter was within the 50% credible interval, the 89% credible interval and how much the estimated median deviates from the ‘true’ parameter values (Table T5). Out of 100 survival estimates, 59 estimates contained the ‘true’ survival value within the 50% credible interval, 92 estimates within the 89% credible interval. The mean deviation from the ‘true’ parameter values was 0.0456± 0.0473 (mean + SD). This indicates our survival estimates are robust, despite the relatively small sample sizes for a CMR study.

Table T5: Results from the simulation study for 50 models, including mean estimate, standard deviation, lower credible interval 89% (LCI89), lower credible interval 50% (LCI50), upper credible interval 50% (UCI50), upper credible interval 89% (UCI89), Gelman-Rubin statistics (Rhat), number of effective sample size (n.eff), the ‘true’ parameter value an optimal model should detect, an assessment if the model could ppredict the ‘true’ value within its 50%CI (in50ci) and 89%CI (in89ci), and how much the model mean estimate deviates from the mean.

model	estimate	mean	sd	LCI89	LCI50	UCI50	UCI89	Rhat	n.eff	true_value	in50ci	in89ci	Deviate from mean
1	phiBP_neg	0.502	0.037	0.442	0.476	0.527	0.562	1	3760	0.458	FALSE	TRUE	0.044
1	phiBP_pos	0.27	0.05	0.195	0.235	0.303	0.352	1	2733	0.235	FALSE	TRUE	0.035
2	phiBP_neg	0.234	0.046	0.164	0.202	0.265	0.31	1	3610	0.261	TRUE	TRUE	0.027
2	phiBP_pos	0.301	0.032	0.25	0.278	0.322	0.353	1	4361	0.31	TRUE	TRUE	0.009
3	phiBP_neg	0.464	0.034	0.41	0.441	0.487	0.519	1	3146	0.441	TRUE	TRUE	0.023
3	phiBP_pos	0.788	0.075	0.665	0.739	0.84	0.904	1	1540	0.795	TRUE	TRUE	0.007
4	phiBP_neg	0.431	0.078	0.311	0.378	0.482	0.56	1	2781	0.485	FALSE	TRUE	0.054
4	phiBP_pos	0.527	0.042	0.462	0.499	0.555	0.594	1	1623	0.56	FALSE	TRUE	0.033
5	phiBP_neg	0.726	0.033	0.675	0.704	0.747	0.778	1	2057	0.694	FALSE	TRUE	0.032
5	phiBP_pos	0.246	0.063	0.151	0.201	0.288	0.353	1	2565	0.246	TRUE	TRUE	0
6	phiBP_neg	0.249	0.045	0.181	0.217	0.279	0.325	1	2168	0.233	TRUE	TRUE	0.016
6	phiBP_pos	0.67	0.055	0.584	0.632	0.707	0.761	1	1537	0.599	FALSE	TRUE	0.071
7	phiBP_neg	0.393	0.093	0.251	0.326	0.453	0.548	1	3317	0.414	TRUE	TRUE	0.021

7	phiBP_pos	0.406	0.032	0.355	0.383	0.427	0.458	1	3215	0.311	FALSE	FALSE	0.095
8	phiBP_neg	0.421	0.056	0.332	0.383	0.458	0.513	1	3212	0.399	TRUE	TRUE	0.022
8	phiBP_pos	0.759	0.035	0.703	0.737	0.783	0.814	1	1728	0.796	FALSE	TRUE	0.037
9	phiBP_neg	0.578	0.047	0.502	0.547	0.61	0.652	1	3529	0.484	FALSE	FALSE	0.094
9	phiBP_pos	0.514	0.035	0.459	0.49	0.538	0.572	1	3735	0.463	FALSE	TRUE	0.051
10	phiBP_neg	0.539	0.087	0.402	0.479	0.598	0.681	1.01	2130	0.407	FALSE	TRUE	0.132
10	phiBP_pos	0.314	0.036	0.258	0.29	0.337	0.374	1	2041	0.35	FALSE	TRUE	0.036
11	phiBP_neg	0.763	0.025	0.723	0.746	0.78	0.803	1	3085	0.782	FALSE	TRUE	0.019
11	phiBP_pos	0.429	0.074	0.313	0.378	0.479	0.548	1	2989	0.338	FALSE	TRUE	0.091
12	phiBP_neg	0.423	0.029	0.377	0.402	0.442	0.469	1	4123	0.415	TRUE	TRUE	0.008
12	phiBP_pos	0.298	0.107	0.141	0.221	0.366	0.482	1	2890	0.247	TRUE	TRUE	0.051
13	phiBP_neg	0.668	0.06	0.575	0.626	0.708	0.765	1.02	612	0.621	FALSE	TRUE	0.047
13	phiBP_pos	0.488	0.081	0.365	0.431	0.541	0.624	1.01	1436	0.418	FALSE	TRUE	0.07
14	phiBP_neg	0.411	0.031	0.362	0.39	0.432	0.462	1	3378	0.414	TRUE	TRUE	0.003
14	phiBP_pos	0.437	0.075	0.319	0.386	0.487	0.56	1	3162	0.437	TRUE	TRUE	0
15	phiBP_neg	0.613	0.111	0.451	0.536	0.678	0.805	1	429	0.491	FALSE	TRUE	0.122
15	phiBP_pos	0.294	0.061	0.201	0.252	0.333	0.396	1	1031	0.309	TRUE	TRUE	0.015
16	phiBP_neg	0.174	0.086	0.058	0.109	0.227	0.328	1	1998	0.299	FALSE	TRUE	0.125
16	phiBP_pos	0.36	0.039	0.304	0.334	0.383	0.424	1	1283	0.404	FALSE	TRUE	0.044
17	phiBP_neg	0.278	0.048	0.207	0.244	0.309	0.361	1	1635	0.365	FALSE	FALSE	0.087
17	phiBP_pos	0.247	0.054	0.163	0.209	0.283	0.336	1	1705	0.218	TRUE	TRUE	0.029
18	phiBP_neg	0.376	0.083	0.249	0.318	0.432	0.513	1.01	2043	0.332	TRUE	TRUE	0.044
18	phiBP_pos	0.452	0.046	0.381	0.42	0.482	0.528	1	1326	0.53	FALSE	FALSE	0.078

19	phiBP_neg	0.594	0.053	0.507	0.558	0.63	0.677	1	3689	0.592	TRUE	TRUE	0.002
19	phiBP_pos	0.287	0.033	0.236	0.264	0.309	0.342	1	3851	0.235	FALSE	FALSE	0.052
20	phiBP_neg	0.725	0.063	0.622	0.682	0.768	0.824	1	1184	0.743	TRUE	TRUE	0.018
20	phiBP_pos	0.451	0.049	0.376	0.417	0.483	0.532	1.01	1450	0.377	FALSE	TRUE	0.074
21	phiBP_neg	0.198	0.027	0.157	0.179	0.216	0.241	1	3832	0.228	FALSE	TRUE	0.03
21	phiBP_pos	0.699	0.095	0.549	0.633	0.763	0.854	1	1076	0.718	TRUE	TRUE	0.019
22	phiBP_neg	0.276	0.103	0.13	0.201	0.341	0.455	1	1829	0.221	TRUE	TRUE	0.055
22	phiBP_pos	0.385	0.044	0.319	0.354	0.413	0.458	1.01	1432	0.37	TRUE	TRUE	0.015
23	phiBP_neg	0.624	0.039	0.562	0.598	0.65	0.685	1	3170	0.715	FALSE	FALSE	0.091
23	phiBP_pos	0.645	0.039	0.582	0.618	0.672	0.707	1	3152	0.67	TRUE	TRUE	0.025
24	phiBP_neg	0.885	0.059	0.787	0.844	0.928	0.977	1	574	0.751	FALSE	FALSE	0.134
24	phiBP_pos	0.369	0.046	0.296	0.338	0.401	0.442	1	1592	0.336	FALSE	TRUE	0.033
25	phiBP_neg	0.384	0.047	0.31	0.352	0.414	0.462	1	2197	0.409	TRUE	TRUE	0.025
25	phiBP_pos	0.539	0.05	0.461	0.505	0.572	0.618	1	2365	0.465	FALSE	TRUE	0.074
26	phiBP_neg	0.398	0.095	0.256	0.329	0.459	0.559	1.01	932	0.443	TRUE	TRUE	0.045
26	phiBP_pos	0.266	0.046	0.197	0.233	0.296	0.342	1	1449	0.227	FALSE	TRUE	0.039
27	phiBP_neg	0.763	0.034	0.708	0.74	0.786	0.816	1	1709	0.759	TRUE	TRUE	0.004
27	phiBP_pos	0.738	0.058	0.646	0.697	0.778	0.831	1.01	1464	0.752	TRUE	TRUE	0.014
28	phiBP_neg	0.631	0.051	0.549	0.596	0.665	0.711	1	3292	0.622	TRUE	TRUE	0.009
28	phiBP_pos	0.332	0.037	0.275	0.307	0.357	0.392	1	3291	0.31	TRUE	TRUE	0.022
29	phiBP_neg	0.557	0.059	0.468	0.517	0.594	0.656	1.01	915	0.49	FALSE	TRUE	0.067
29	phiBP_pos	0.406	0.065	0.307	0.362	0.448	0.512	1	1416	0.496	FALSE	TRUE	0.09
30	phiBP_neg	0.083	0.081	0.005	0.026	0.115	0.231	1.04	679	0.247	FALSE	FALSE	0.164

30	phiBP _pos	0.592	0.059	0.521	0.557	0.615	0.68	1.02	467	0.558	TRUE	TRUE	0.034
31	phiBP _neg	0.148	0.077	0.045	0.089	0.194	0.289	1	2126	0.325	FALSE	FALSE	0.177
31	phiBP _pos	0.773	0.03	0.726	0.753	0.793	0.821	1	1816	0.736	FALSE	TRUE	0.037
32	phiBP _neg	0.382	0.053	0.305	0.344	0.416	0.475	1	760	0.429	FALSE	TRUE	0.047
32	phiBP _pos	0.672	0.094	0.522	0.607	0.736	0.823	1	1000	0.677	TRUE	TRUE	0.005
33	phiBP _neg	0.537	0.048	0.462	0.504	0.568	0.615	1	2839	0.53	TRUE	TRUE	0.007
33	phiBP _pos	0.598	0.037	0.54	0.573	0.622	0.657	1	3561	0.6	TRUE	TRUE	0.002
34	phiBP _neg	0.615	0.092	0.485	0.55	0.67	0.777	1.01	362	0.517	FALSE	TRUE	0.098
34	phiBP _pos	0.408	0.064	0.311	0.363	0.449	0.516	1.01	1218	0.373	TRUE	TRUE	0.035
35	phiBP _neg	0.679	0.065	0.585	0.634	0.717	0.792	1.01	596	0.716	TRUE	TRUE	0.037
35	phiBP _pos	0.243	0.057	0.159	0.203	0.279	0.336	1	1812	0.247	TRUE	TRUE	0.004
36	phiBP _neg	0.353	0.043	0.286	0.323	0.381	0.424	1	1822	0.379	TRUE	TRUE	0.026
36	phiBP _pos	0.708	0.069	0.6	0.663	0.753	0.822	1	1217	0.676	TRUE	TRUE	0.032
37	phiBP _neg	0.208	0.04	0.15	0.18	0.232	0.276	1	1679	0.217	TRUE	TRUE	0.009
37	phiBP _pos	0.249	0.077	0.138	0.194	0.295	0.382	1	1659	0.221	TRUE	TRUE	0.028
38	phiBP _neg	0.798	0.056	0.711	0.759	0.835	0.891	1.01	657	0.772	TRUE	TRUE	0.026
38	phiBP _pos	0.519	0.053	0.435	0.484	0.554	0.604	1.01	1666	0.479	FALSE	TRUE	0.04
39	phiBP _neg	0.274	0.036	0.219	0.248	0.298	0.333	1	2995	0.275	TRUE	TRUE	0.001
39	phiBP _pos	0.273	0.06	0.183	0.23	0.312	0.377	1	2468	0.417	FALSE	FALSE	0.144
40	phiBP _neg	0.583	0.038	0.522	0.557	0.608	0.645	1	3376	0.617	FALSE	TRUE	0.034
40	phiBP _pos	0.574	0.042	0.506	0.546	0.602	0.641	1	3511	0.499	FALSE	FALSE	0.075
41	phiBP _neg	0.271	0.034	0.216	0.247	0.293	0.326	1	4163	0.269	TRUE	TRUE	0.002
41	phiBP _pos	0.274	0.044	0.206	0.244	0.303	0.346	1	3567	0.314	FALSE	TRUE	0.04

42	phiBP_neg	0.37	0.065	0.269	0.323	0.413	0.478	1	1062	0.243	FALSE	FALSE	0.127
42	phiBP_pos	0.424	0.076	0.308	0.371	0.474	0.55	1.01	893	0.514	FALSE	TRUE	0.09
43	phiBP_neg	0.252	0.039	0.193	0.225	0.277	0.316	1	2164	0.219	FALSE	TRUE	0.033
43	phiBP_pos	0.53	0.076	0.413	0.477	0.581	0.656	1.01	1538	0.511	TRUE	TRUE	0.019
44	phiBP_neg	0.648	0.041	0.585	0.62	0.674	0.714	1	1776	0.601	FALSE	TRUE	0.047
44	phiBP_pos	0.395	0.072	0.28	0.345	0.443	0.512	1	2121	0.334	FALSE	TRUE	0.061
45	phiBP_neg	0.447	0.069	0.34	0.4	0.492	0.561	1	1554	0.396	FALSE	TRUE	0.051
45	phiBP_pos	0.451	0.054	0.365	0.414	0.486	0.54	1	1164	0.479	TRUE	TRUE	0.028
46	phiBP_neg	0.289	0.034	0.238	0.266	0.311	0.346	1	4333	0.254	FALSE	TRUE	0.035
46	phiBP_pos	0.268	0.043	0.202	0.237	0.295	0.339	1	2548	0.254	TRUE	TRUE	0.014
47	phiBP_neg	0.512	0.04	0.45	0.486	0.539	0.577	1	4009	0.541	FALSE	TRUE	0.029
47	phiBP_pos	0.355	0.038	0.294	0.329	0.381	0.416	1	4246	0.372	TRUE	TRUE	0.017
48	phiBP_neg	0.507	0.033	0.454	0.485	0.529	0.559	1	3610	0.519	TRUE	TRUE	0.012
48	phiBP_pos	0.732	0.057	0.641	0.694	0.772	0.822	1	2841	0.728	TRUE	TRUE	0.004
49	phiBP_neg	0.556	0.063	0.459	0.513	0.598	0.658	1	2223	0.57	TRUE	TRUE	0.014
49	phiBP_pos	0.26	0.04	0.201	0.233	0.285	0.326	1	1994	0.263	TRUE	TRUE	0.003
50	phiBP_neg	0.284	0.038	0.224	0.258	0.309	0.345	1	3986	0.269	TRUE	TRUE	0.015
50	phiBP_pos	0.405	0.04	0.343	0.377	0.431	0.47	1	3728	0.398	TRUE	TRUE	0.007

References

1. LfU Brandenburg. CIR (colour-infrared)-Biotope types 2009. (2009).
2. Hijmans, R. J., Bivand, R., Pebesma, E. & Sumner, M. D. terra: Spatial Data Analysis. (2023).
3. Wei, T. & Simko, V. *R Package 'Corrplot': Visualization of a Correlation Matrix*. (2021).
4. Hellgren, O., Waldenström, J. & Bensch, S. A new PCR assay for simultaneous studies of Leucocytozoon, Plasmodium, and Haemoproteus from avian blood. *J Parasitol* **90**, 797–802 (2004).
5. Çakmak, E., Akın Pekşen, Ç. & Bilgin, C. C. Comparison of three different primer sets for sexing birds. *J VET Diagn Invest* **29**, 59–63 (2017).
6. Lee, J. C.-I. *et al.* A novel strategy for avian species and gender identification using the CHD gene. *Molecular and Cellular Probes* **24**, 27–31 (2010).
7. Pebesma, E. Simple Features for R: Standardized Support for Spatial Vector Data. *The R Journal* **10**, 439 (2018).
8. Pebesma, E. & Bivand, R. *Spatial Data Science: With Applications in R*. (Chapman and Hall/CRC, New York, 2023). doi:10.1201/9780429459016.
9. Wickham, H. *et al.* dplyr: A Grammar of Data Manipulation. (2023).
10. Thieurmel & Elmarhraoui. suncalc: Compute Sun Position, Sunlight Phases, Moon Position and Lunar Phase. (2022).
11. Calabrese, J. M., Fleming, C. H. & Gurarie, E. ctmm: an R package for analyzing animal relocation data as a continuous-time stochastic process. *Methods Ecol Evol* **7**, 1124–1132 (2016).
12. Beardsworth, C. E. *et al.* Validating a high-throughput tracking system: ATLAS as a regional-scale alternative to GPS 2. *bioRxiv* 2021.02.09.430514 (2021).
13. Winner, K. *et al.* Statistical inference for home range overlap. *Methods in Ecology and Evolution* **9**, 1679–1691 (2018).

14. Avgar, T., Potts, J. R., Lewis, M. A. & Boyce, M. S. Integrated step selection analysis: bridging the gap between resource selection and animal movement. *Methods in Ecology and Evolution* **7**, 619–630 (2016).

15. Kassambara, A. & Mundt, F. *Factoextra: Extract and Visualize the Results of Multivariate Data Analyses*. (2020).
