# Supplementary material for: Sick without signs. Subclinical infections reduce local movements, alter habitat selection, and cause demographic shifts
Source: Commun Biol. 2024 Nov 1;7:1426. doi: 10.1038/s42003-024-07114-4 (PMC11530534; doi:10.1038/s42003-024-07114-4)
Supplement: Supplementary file 3 — Description of Additional Supplementary File [file 42003_2024_7114_MOESM3_ESM.pdf]

Description Of Additional Supplementary File

File name: Supplementary Movie S1

Description: Exemplary movement tracks Description: Animation to show movement data of swallows during foraging events in the study area. Each dot represents an individual swallow on a foraging trip, and each line indicates the suggested path between two recorded points. Due to computational constraints, the original tracking data were resampled from 8-second recordings (0.125Hz) to 40-second intervals. Note: Resampling increases the temporal resolution by a factor of five, displayed here solely for illustrative purposes.
