# Supplementary figures and images for: Sick without signs. Subclinical infections reduce local movements, alter habitat selection, and cause demographic shifts
Source: Commun Biol. 2024 Nov 1;7:1426. doi: 10.1038/s42003-024-07114-4 (PMC11530534; doi:10.1038/s42003-024-07114-4)

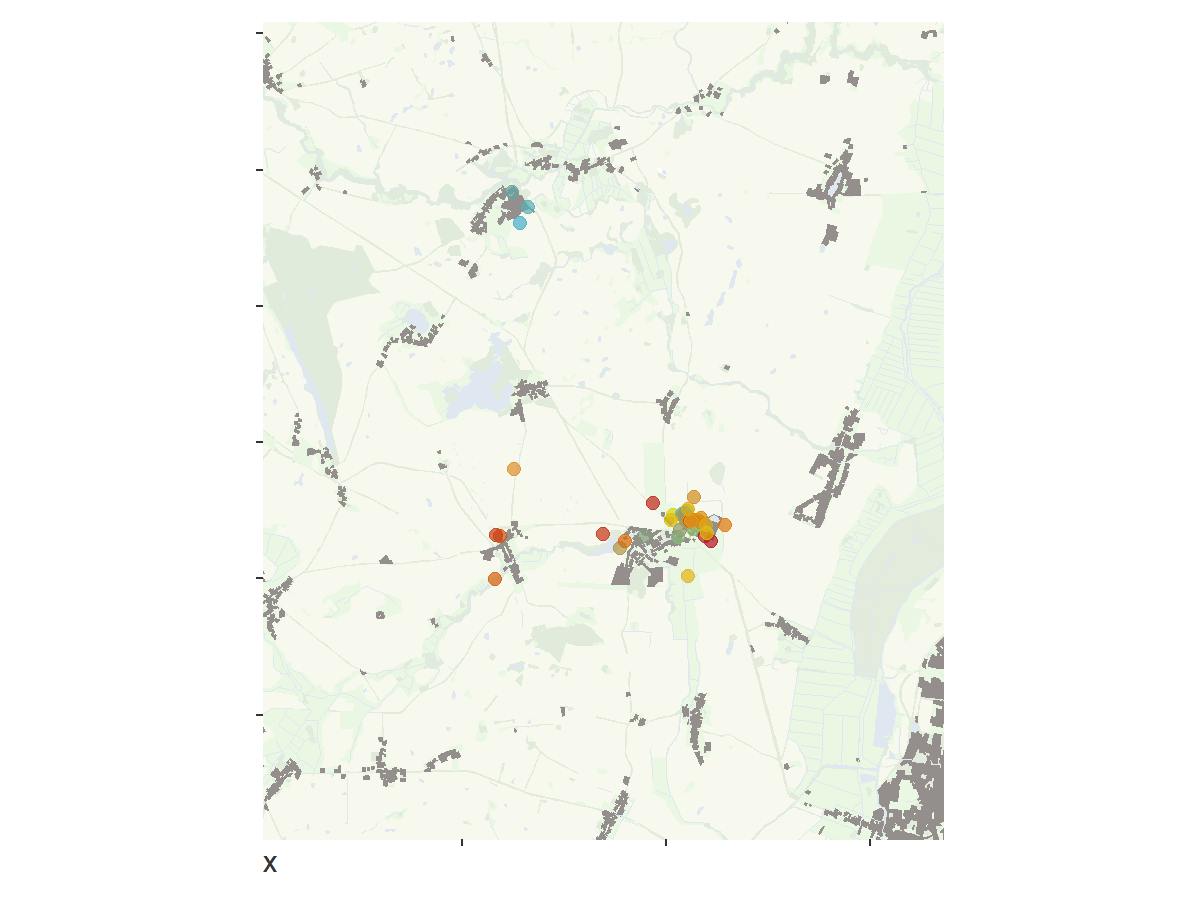

Supplement: Supplementary file 4 — Supplementary Movie S1 [file 42003_2024_7114_MOESM4_ESM.gif]
