# Supplementary material for: Sick without signs. Subclinical infections reduce local movements, alter habitat selection, and cause demographic shifts
Source: Commun Biol. 2024 Nov 1;7:1426. doi: 10.1038/s42003-024-07114-4 (PMC11530534; doi:10.1038/s42003-024-07114-4)
Supplement: Supplementary file 5 — Reporting summary [file 42003_2024_7114_MOESM5_ESM.pdf]

nature portfolio

Corresponding author(s): Marius Grabow; grabow@izw-berlin.de

Last updated by author(s): Oct 25, 2024

# Reporting Summary

Nature Portfolio wishes to improve the reproducibility of the work that we publish. This form provides structure for consistency and transparency in reporting. For further information on Nature Portfolio policies, see our Editorial Policies and the Editorial Policy Checklist.

## Statistics

For all statistical analyses, confirm that the following items are present in the figure legend, table legend, main text, or Methods section.

| n/a | Confirmed | |
|---|---|---|
| ☐ | ☒ | The exact sample size (*n*) for each experimental group/condition, given as a discrete number and unit of measurement |
| ☐ | ☒ | A statement on whether measurements were taken from distinct samples or whether the same sample was measured repeatedly |
| ☐ | ☒ | The statistical test(s) used AND whether they are one- or two-sided<br>*Only common tests should be described solely by name; describe more complex techniques in the Methods section.* |
| ☐ | ☒ | A description of all covariates tested |
| ☐ | ☒ | A description of any assumptions or corrections, such as tests of normality and adjustment for multiple comparisons |
| ☐ | ☒ | A full description of the statistical parameters including central tendency (e.g. means) or other basic estimates (e.g. regression coefficient) AND variation (e.g. standard deviation) or associated estimates of uncertainty (e.g. confidence intervals) |
| ☐ | ☒ | For null hypothesis testing, the test statistic (e.g. *F*, *t*, *r*) with confidence intervals, effect sizes, degrees of freedom and *P* value noted<br>*Give P values as exact values whenever suitable.* |
| ☐ | ☒ | For Bayesian analysis, information on the choice of priors and Markov chain Monte Carlo settings |
| ☐ | ☒ | For hierarchical and complex designs, identification of the appropriate level for tests and full reporting of outcomes |
| ☐ | ☒ | Estimates of effect sizes (e.g. Cohen's *d*, Pearson's *r*), indicating how they were calculated |

*Our web collection on statistics for biologists contains articles on many of the points above.*

## Software and code

Policy information about availability of computer code

| | |
|---|---|
| Data collection | no software was used for capture-recapture data collection. ATLAS (Toledo et al. 2020) was used to collect movement data. |
| Data analysis | We performed all statistical analyses using R 4.2.1, and utilized the NIMBLE programming language via R 4.2.1 for all MCMC / bayesian methods. |

For manuscripts utilizing custom algorithms or software that are central to the research but not yet described in published literature, software must be made available to editors and reviewers. We strongly encourage code deposition in a community repository (e.g. GitHub). See the Nature Portfolio guidelines for submitting code & software for further information.

## Data

Policy information about availability of data

All manuscripts must include a data availability statement. This statement should provide the following information, where applicable:

- Accession codes, unique identifiers, or web links for publicly available datasets
- A description of any restrictions on data availability
- For clinical datasets or third party data, please ensure that the statement adheres to our policy

The movement data used for this study is contained in a movebank repository and available on request (Movebank ID: 3053965481). The capture-recapture data will be publicly available upon publication in a Dryad data repository.

## Research involving human participants, their data, or biological material

Policy information about studies with human participants or human data. See also policy information about sex, gender (identity/presentation), and sexual orientation and race, ethnicity and racism.

| | |
|---|---|
| Reporting on sex and gender | NA |
| Reporting on race, ethnicity, or other socially relevant groupings | NA |
| Population characteristics | NA |
| Recruitment | NA |
| Ethics oversight | NA |

Note that full information on the approval of the study protocol must also be provided in the manuscript.

# Field-specific reporting

Please select the one below that is the best fit for your research. If you are not sure, read the appropriate sections before making your selection.

☐ Life sciences ☐ Behavioural & social sciences ☒ Ecological, evolutionary & environmental sciences

For a reference copy of the document with all sections, see nature.com/documents/nr-reporting-summary-flat.pdf

# Ecological, evolutionary & environmental sciences study design

All studies must disclose on these points even when the disclosure is negative.

| | |
|---|---|
| Study description | We studied movement behaviour & demography, particularly survival, of non-infected and infected individuals of two sympatric swallow species. |
| Research sample | Two populations of sympatric swallow species, breeding in the same dairy farm in northeast Germany (N 53.38°, E 13.75°) close to the city of Prenzlau. Swallows were chosen as a study species because their distinct flight patterns are detected reliably by the ATLAS system that requires line-of-sight. Moreover, they perform long distance migrations that potentially exposes them to more pathogens / parasites, allowing rather balanced study design. |
| Sampling strategy | Individuals that fulfilled the ethic permit requirements (i.e. a minimum weight og 19g in barn swallows, and 20g in house martins) were tagged with ATLAS tags. Blood samples were collected for all individuals when possible. In few cases, medical condition, blood sampling procedure, or laboratory procedures prevented assessing the infection status. |
| Data collection | Movement data was collected automatically. Birds of both species were captured using mist nets (Ecotone 1014/3, mesh 14 × 14 mm). For blood sampling, we punctured their brachial vein with a hollow needle. We collected small amounts of blood (approx. 30µl), stored it in stabilization buffer (Qiagen RNAprotect) and later extracted DNA and RNA to assess blood parasite and Flavivirus infection, respectively |
| Timing and spatial scale | We applied a structured capture-mark-recapture (CMR) design, in which each primary occasion (year) consisted of two secondary sampling occasions exactly 14 days apart (21st and 23rd calendar week). We minimized capturing biases by placing mist nests directly at the gates of the dairy farms (standardized capturing procedure, 7 hours per capturing event) and maintained an equal sampling intensity. |
| Data exclusions | Infection status could not be collected for all individuals, but no animals were excluded. We excluded tagged individuals with tag failures and very short tracking durations, i.e. only those were the technology or attachment mehtod failed. |
| Reproducibility | This is a correlative study. We could observe the same effects across multiple breeding seasons |
| Randomization | This is a correlative study. There was no randomization |
| Blinding | This is a correlative study. There was no blinding |

Did the study involve field work? ☒ Yes ☐ No

## Field work, collection and transport

| | |
|---|---|
| Field conditions | Field work was conducted within a structured capture-mark-recapture study. Capturing dates, sampling efforts, and locations were kept constant across the sampling period. Swallow nests were located inside a large dairy farm, protecting them from harsh climatic conditions, and allowing for almost identical conditions across years |
| Location | We conducted our study in northeast Germany (N 53.38°, E 13.75°) close to the city of Prenzlau |
| Access & import/export | Swallows breed inside a large dairy farm, allowing for easy access. All animal handling procedures adhered to the protocols no. 2347-22-2019, as approved by the Brandenburg National Office for Occupational Safety, Consumer Protection and Health, and Beri-017b-21, as approved by the Brandenburg National Office for the Environment. |
| Disturbance | We disturbed animals by mist-netting. We minimized the disturbances by following good practices of mist-netting, further accounted for that disturbances and removed the initial hours of movement data from all movement models. |

# Reporting for specific materials, systems and methods

We require information from authors about some types of materials, experimental systems and methods used in many studies. Here, indicate whether each material, system or method listed is relevant to your study. If you are not sure if a list item applies to your research, read the appropriate section before selecting a response.

### Materials & experimental systems

| n/a | Involved in the study | |
|---|---|---|
| ☒ | ☐ | Antibodies |
| ☒ | ☐ | Eukaryotic cell lines |
| ☒ | ☐ | Palaeontology and archaeology |
| ☐ | ☒ | Animals and other organisms |
| ☒ | ☐ | Clinical data |
| ☒ | ☐ | Dual use research of concern |
| ☒ | ☐ | Plants |

### Methods

| n/a | Involved in the study | |
|---|---|---|
| ☒ | ☐ | ChIP-seq |
| ☒ | ☐ | Flow cytometry |
| ☒ | ☐ | MRI-based neuroimaging |

## Animals and other research organisms

Policy information about studies involving animals; ARRIVE guidelines recommended for reporting animal research, and Sex and Gender in Research

| | |
|---|---|
| Laboratory animals | The study did not involve laboratory animals |
| Wild animals | Two species of wild animals were captured: Barn swallows (Hirundo rustica) and House martins (Delichon urbicum). Birds of both species were captured using mist nets (Ecotone 1014/3, mesh 14 × 14 mm). Animals were released after capturing, blood sampling, and tagging (<10 min handling time). All animals were released at their capturing location. |
| Reporting on sex | We tested for sex-specific effects but found no difference in effect sizes or direction in both species. |
| Field-collected samples | We collected small amounts of blood (approx. 30µl), stored it in stabilization buffer (Qiagen RNAprotect). Following the instructions of the manufacturer of the stabilization buffer, we first stored samples for ~ 2 hours at room temperature (15-22 ° Celcius) to ensure efficient lysis, then froze samples at -20°C for transport, and at -80°C for storage. |
| Ethics oversight | ll animal handling procedures adhered to the protocols no. 2347-22-2019, as approved by the Brandenburg National Office for Occupational Safety, Consumer Protection and Health, and Beri-017b-21, as approved by the Brandenburg National Office for the Environment. |

Note that full information on the approval of the study protocol must also be provided in the manuscript.

## Plants

| | |
|---|---|
| Seed stocks | NA |
| Novel plant genotypes | NA |
| Authentication | NA |
